# Case Report: Resting-State Brain-Networks After Near-Complete Hemispherectomy in Adulthood

**DOI:** 10.3389/fneur.2022.885115

**Published:** 2022-06-10

**Authors:** Patrick M. Fisher, Simon S. Albrechtsen, Vardan Nersesjan, Moshgan Amiri, Daniel Kondziella

**Affiliations:** ^1^Department of Neurology, Rigshospitalet, Copenhagen University Hospital, Copenhagen, Denmark; ^2^Neurobiology Research Unit, Rigshospitalet, Copenhagen University Hospital, Copenhagen, Denmark; ^3^Department of Clinical Medicine, University of Copenhagen, Copenhagen, Denmark

**Keywords:** functional MRI, brain networks, cognition, consciousness, brain injury, stroke, cerebral infarction, malignant middle cerebral artery infarction

## Abstract

**Objectives:**

Understanding the dynamics of reorganized network-level brain functions after hemispherectomy is important for treatment, prognostication, and rehabilitation of brain injury, but also for investigating questions of fundamental neurobehavioral interest: How does the brain promote consciousness despite loss of one hemisphere?

**Methods:**

We studied resting-state functional connectivity (RSFC) in a high-functioning middle-aged man 6 years after functional hemispherectomy following malignant middle cerebral artery infarction, and we compared results to RSFC in 20 healthy controls.

**Results:**

Our analysis indicates increased between-network connectivity for all seven networks examined in the patient's preserved hemisphere, compared to healthy controls, suggesting a shift toward increased between-network connectivity following near-complete loss of one hemisphere during adulthood.

**Conclusions:**

These data corroborate and extend recent findings of increased between-network connectivity in the remaining hemisphere after surgical hemispherectomy for intractable epilepsy during childhood. Our results support a neuroplasticity model with reorganization of distributed brain connectivity within the preserved hemisphere as part of the road to recovery after brain injury, as well as recovery of consciousness and cognitive functions, after hemispherectomy.

## Introduction

Following middle cerebral artery (MCA) stroke, cerebral edema and brain tissue herniation may lead to rapid neurological deterioration and death, which is known as malignant MCA infarction. Clinical outcomes with emergency hemicraniectomy vary widely (1). Characterizing retained and altered systems-level neural function is of neurobiological interest and important for treatment, prognostication, and rehabilitation.

Resting-state fMRI (rs-fMRI) with blood-oxygen level dependent (BOLD) brain imaging is used to estimate resting-state functional connectivity (RSFC), a measure of the correlation strength in BOLD signal between discrete brain regions. Rs-fMRI has been used to characterize sets of regions, known as resting-state networks, which are thought to support consciousness, cognition and behavior.

A recent study in six adults who underwent surgical hemispherectomy during childhood for intractable epilepsy reported that RSFC within seven brain networks was generally intact in the preserved hemisphere, but RSFC between networks was substantively increased compared to healthy controls (2). Moreover, increased contralateral and inter-hemispheric connectivity have been reported in a previous study employing rs-fMRI in complete surgical hemispherotomy, including total callosotomy with a perithalamic section, in two children (3). This suggests that hemispherectomy results in a strengthening of preserved inter-network connectivity, despite major structural disruptions, and shows that rs-fMRI can inform alterations in brain function in patients after hemispherectomy in childhood. By contrast, it is unclear if similar RSFC characteristics can be observed in patients who undergo hemispherectomy, whether surgically or functionally following malignant MCA infarction, in adulthood.

Here we investigated RSFC in a high-functioning adult man with malignant MCA infarction 6 years earlier, resulting in near-total loss of the right hemisphere, compared to RSFC in 20 healthy controls.

## Methods

Following written informed consent, a 45-year-old man was recruited for clinical and neuropsychological evaluation to assess the degree of neurocognitive deficits and brain imaging with fMRI to investigate RSFC. He was recruited within the CONNECT-ME study (ClinicalTrials.gov Identifier: NCT02644265) (4).

### Clinical and Neuropsychological Evaluation

A full neurological evaluation including National Institutes of Health Stroke Scale (NIHSS) (5) and the following cognitive and neuropsychological tests were performed: Montreal Cognitive Assessment (6), Frontal Assessment Battery (7), Block Design Test (8), Street's Completion Test (9, 10), and the Star Cancellation Test (11).

### fMRI Data Acquisition

All MRI data were acquired at Rigshospitalet, Copenhagen University Hospital, on a Siemens (Erlangen, DE) MAGNETOM 3T Prisma scanner with a 32-channel head coil. High-resolution, whole-brain, T1-weighted MPRAGE structural scans were acquired (inversion time = 972 msec, repetition time = 2,000 msec, echo time = 2.58 msec, flip angle = 8°, in-plane matrix = 256 × 256 mm, in-plane resolution = 0.9 × 0.9 mm, 224 slices, slice thickness = 0.9 mm). Blood-oxygen level dependent (BOLD) fMRI scans were acquired using a T2^*^-weighted gradient echo-planar imaging (EPI) sequence (repetition time = 2,000 msec, echo time = 30 msec, flip angle = 70°, in-plane matrix = 76 × 76 mm, in-plane resolution = 3 × 3 mm, 35 slices (acquired interleaved, bottom-up), slice thickness = 3 mm, gap = 0.6 mm, total volumes = 300, scan time = 10 min). During resting-state scan sessions, participants were asked to close their eyes, let their mind wander and to not fall asleep. A corresponding field map was acquired to unwarp spatial distortions in EPI images.

Resting-sate fMRI data were pre-processed using Statistical Parametric Mapping 12 (https://www.fil.ion.ucl.ac.uk/spm/software/spm12/). Functional images were corrected for slice-timing, spatially realigned, corrected for spatial distortions and co-registered to the high-resolution structural image. The high-resolution structural image was normalized into Montreal Neurologic Institute (MNI) standard space and the corresponding warping map applied to the functional images. Normalized functional images (voxel size: 2 mm isotropic) were smoothed with an 8 mm full-width half-maximum (FWHM) Gaussian filter. Additional denoising of time-series data was performed using CONN (version 19.c) (12). Time series were filtered using a bandpass filter from 0.008 to 0.09 Hz. Additionally, we performed an estimation of physiological noise sources using anatomical component correction (aCompCor): regressing out the time series (and first derivative) of the first five principal components from a decomposition of the time series from white-matter and cerebrospinal fluid voxels, separately. Additionally, we regressed the time series for the six motion parameters (and first derivatives) (13). Mean denoised time series were extracted from regions of interest (ROIs) for further analysis. We calculated the between-region correlation across the entire time series; Pearson's rho correlation estimates were transformed using Fisher's *r*-to-*z* transform [i.e., r-to-z = 0.5 × (ln((1+r)/(1-r))], where *r* is the Pearson's rho and *ln* represents taking the natural logarithm). These *r*-to-*z* values were used to estimate within- and between-network connectivity strength. A previously described 400-region, seven-network atlas was used to determine within- and between-networks (14). The seven networks are: Default Mode, Frontoparietal, Limbic, Salience-Ventral-Attention, Dorsal-Attention, Somatomotor, and Visual. Due to this particular patient case, only left hemisphere regions were considered. Connectivity estimates are similar to those reported previously (2). Within-network connectivity was calculated as the average connectivity strength between all pairs of regions within a specific network. Between-network connectivity was calculated as the average connectivity strength between all pairs of regions from a pair of networks where any pair included one region from each of the two networks. “Composite” between-network connectivity was calculated as the average connectivity strength between all network-specific regions and all regions not belonging to that network. The mean and standard deviation of within- and between-network connectivities within the left hemisphere (preserved in the patient) were computed for the reference population; patient connectivity estimates were expressed as relative z-scores [i.e., (conn_patient_ – mean[conn_ref_])/sd[conn_ref_]].

### Healthy Controls

Rs-fMRI data from 20 healthy controls (median age 27.6 years, range 21.3–55.5 years; 11 males) recruited through on-going and unrelated research studies were included as a reference population to describe differences in patient connectivity. Healthy controls completed identical imaging sequences as the patient, on the same MRI scanner and within 6 months of the patient being scanned. All connectivity estimates were assessed for the left hemisphere. Imaging and data acquisition in these healthy controls was performed after obtaining written informed consent for ethics committee approved studies.

## Results

### Clinical Summary

**Figure 1:**
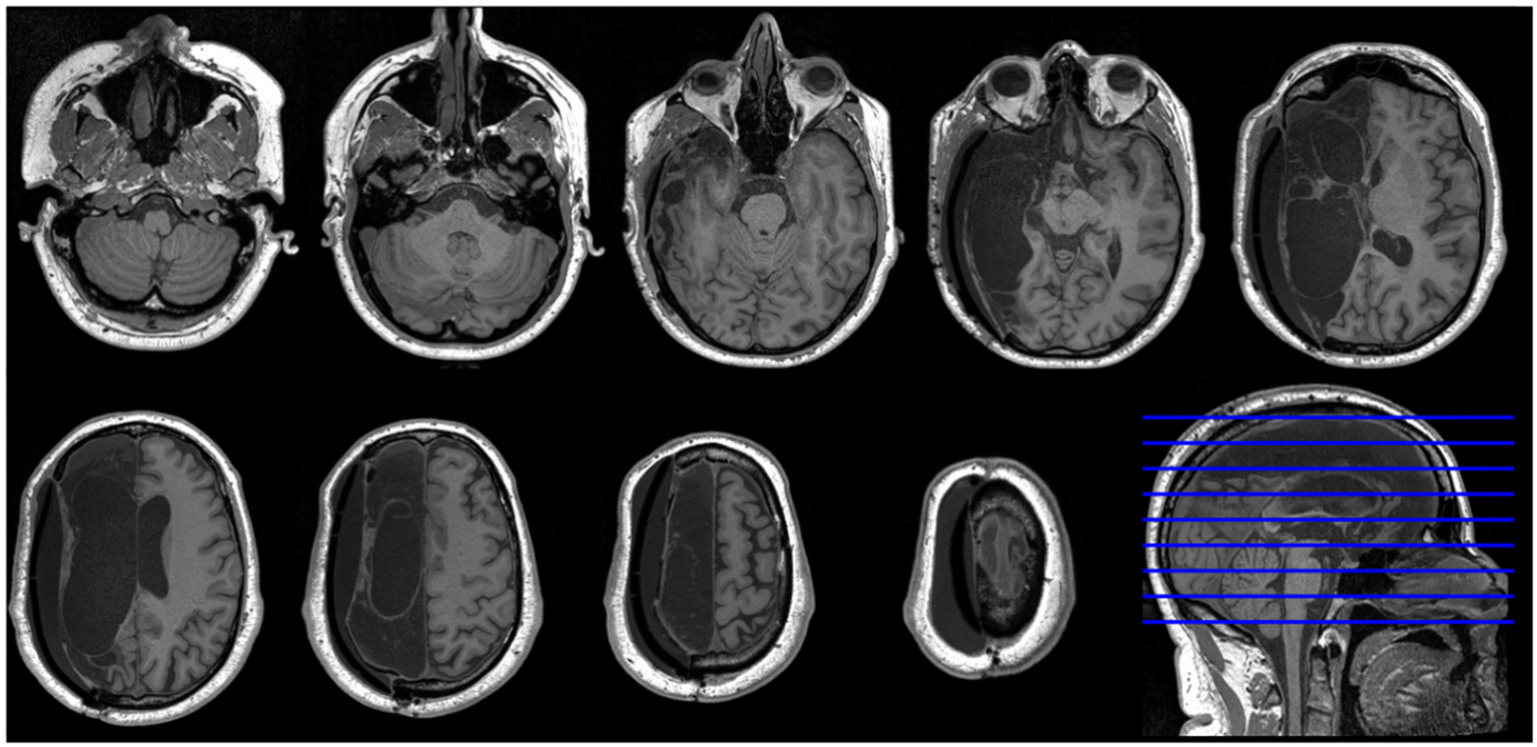
High-resolution axial MRI slices of patient brain. Nine axial T1-weighted MRI slices and one sagittal slice of the patient's brain show near-complete loss of right hemispheric brain tissue. According to radiological convention, the left hemisphere is shown on the right side of the figure.

In 2014, at the age of 39 years, a previously healthy male was admitted with left-sided facial palsy, hemianopsia, and hemiparalysis and right-sided gaze deviation (NIHSS score 17), due to a right-sided internal carotid artery occlusion caused by a cardioembolic stroke and atrial fibrillation. Despite endovascular thrombectomy, the patient developed decreased consciousness [Glasgow Coma Scale (15): 5] owing to malignant MCA infarction. Emergency decompressive craniectomy was performed, followed by cranioplasty 3 months later. Two years later, the patient had stable deficits including a left-sided hemianopsia and spastic hemiparesis. He could walk with a cane. Language and orientation were preserved. After 3 years, he resumed part-time work as a graphical designer. MRI showed near-complete loss of right hemispheric brain tissue sparing only parts of the mesial occipital lobe, the inferior pre-cuneus, the isthmus of the cingulate gyrus, and the inferior mesial temporal lobe (Figure 1).

### Clinical Exam

The patient (45-year-old, in 2020) was fully alert and oriented, and his speech was fluent. There was no aphasia, apraxia, or hemispatial neglect. He had left-sided hemianopsia and upper motor neuron hemiparesis, but well-preserved gait function. Neuropsychological testing was within the low normal range, except for the Street's Completion Test (8/20; mean normal score = 13, SD = 3) indicating impaired visuospatial processing (Table 1).

**Table 1:** Clinical, cognitive, and visuospatial evaluation of the patient, 6 years after a right-sided malignant middle cerebral artery infarct resulting in near-complete right hemispherectomy.

**Test**	**Score (total)**	**Specified test results**
National Institutes of Health Stroke Scale (5)	6	Hemianopsia 2; facial palsy 1; arm paresis 2; leg paresis 1
Montreal cognitive assessment (6)	26 (30)	Visuospatial 2/5; naming 3/3; attention 6/6; language 3/3; abstraction 2/2; delayed recall 4/5; orientation 6/6
Frontal assessment battery (7)	17 (18)	Conceptualization 3/3; mental flexibility 3/3; motor series 3/3; conflicting instructions 3/3; no-no go 2/3; prehension behavior 3/3
Block design test (8)	28 (48)	Reaching step 10; scoring 0 on steps 11 and 12, thus ending the session
Street's completion test (9, 10)	8 (20)	Recognizing images 1–4 and 7, 9, and 13
Star cancellation test (11)	53 (54)	Completed the test in 2 min and 40 s

### Brain Connectivity Compared to Healthy Controls

**Figure 2:**
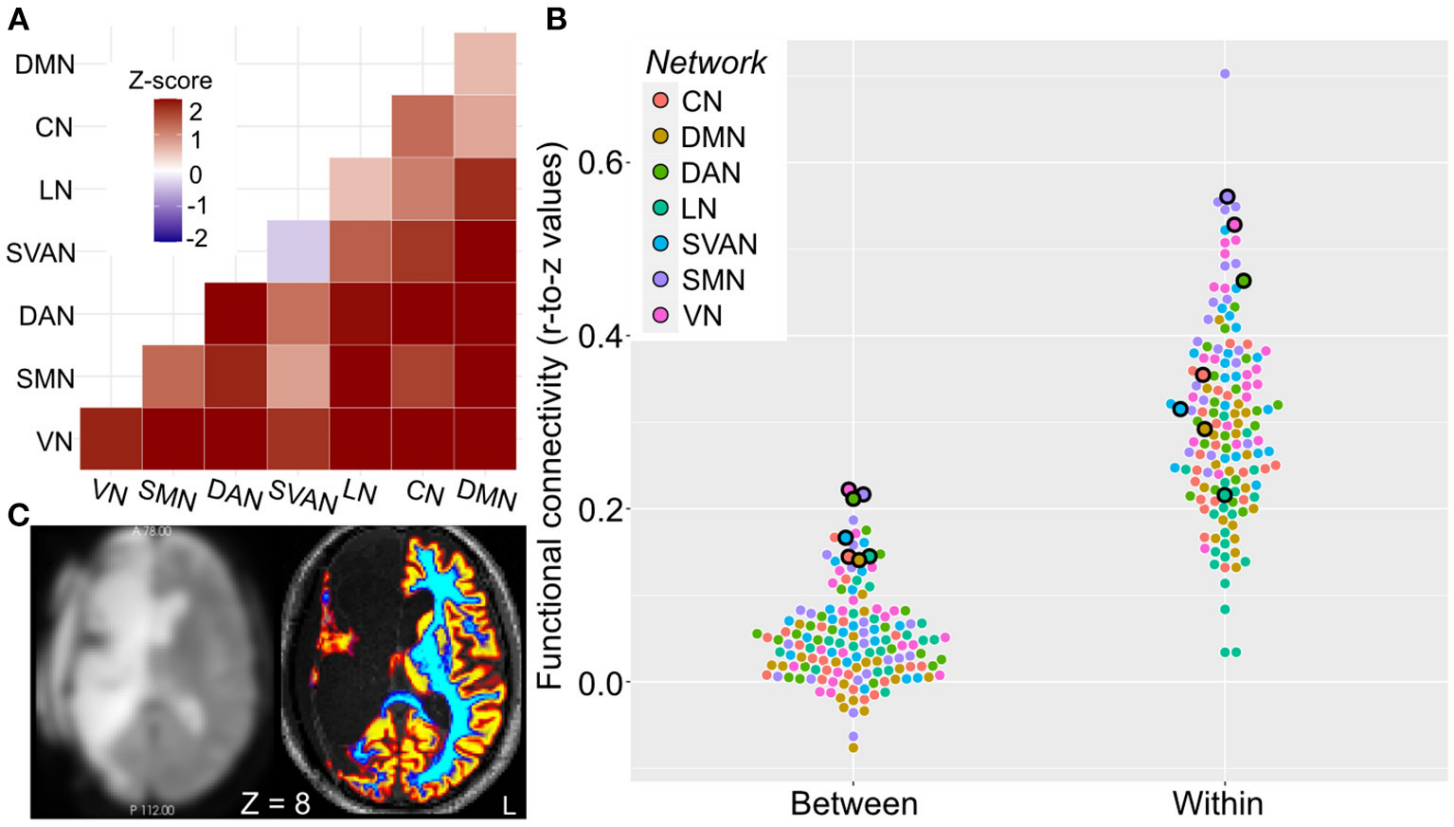
Elevated patient resting-state functional connectivity. Network functional connectivity elevated in the case relative to healthy controls. **(A)** Within- (diagonal squares) and particularly between-network (off-diagonal squares) functional connectivity is elevated in the patient, as indicated by heatmap of z-scores of connectivity in patient relative to healthy controls. **(B)** Mean within- and “composite” between-network functional connectivity for the patient (outlined dots) and healthy controls (dots without outline). Networks are color-coded as illustrated. **(C)** Axial image of preprocessed BOLD fMRI (left) and high-resolution structural (right) images. Coloring indicates successful segmentation across the preserved brain areas (primarily left hemisphere); warm colors denote brain areas with high probability of gray matter, cool colors denote brain areas with high probability of white matter. *Z* is given in MNI coordinates. Left hemisphere is shown on right side of the figure. CN, control network; DAN, dorsal attention network; DMN, default mode network; HC, healthy controls; LN, language network; MNI, montreal national institue; SMN, somatomotor network; SVAN, salience-ventral-attention network; VN, visual network.

Despite loss of brain tissue, image processing, including segmentation, was successful (Figure 2). Compared to the left hemispheres of 20 healthy controls, rs-fMRI of the preserved (i.e., left) hemisphere in the patient showed that within- and between-network resting-state functional connectivity was increased across all networks, except for the Salience-Ventral-Attention network where within-network connectivity was slightly decreased (*z*-score = −0.4; Figures 2A,B). The patient's between-network connectivity was elevated (mean *z*-score = 2.0) for each of the seven networks (Figure 2A). Between-network connectivity including the default mode network and visual network were particularly elevated. The patient had the three highest and seven of the 16 highest individual network “composite” between-network connectivity estimates relative to the healthy controls (Figures 2A–C).

## Discussion

RSFC analysis in this patient with remarkable clinical recovery 6 years after near-complete loss of the right hemisphere indicates increased between-network connectivity for all seven networks examined in the preserved (dominant) hemisphere. This is numerically consistent with two recent reports of eight patients who had surgical hemispherectomy in childhood (2, 3). This suggests that a shift toward increased between-network connectivity can be observed not only following childhood surgical hemispherectomy due to epilepsy, but also in an adult patient within a few years after functional hemispherectomy due to ischemic stroke. Although these data do not establish causality, our case study is consistent with a model wherein a reorganization of distributed brain connectivity within the preserved hemisphere supports good recovery.

We cannot know if these characteristics generalize to all or most patients who recover from malignant MCA infarction, and the temporal development of these changes remain to be discovered. It might be that increased between-network connectivity develops instantaneously after the stroke or gradually with improved neurological function. In addition, it could be hypothesized that people with greater than average a priori between-network connectivity have increased cerebral reserves and better potential for neurological recovery after hemispherectomy, although recent data from adult patients with hemispherectomy during childhood suggest otherwise (2).

The findings provide intriguing evidence suggesting alterations in distributed brain connectivity within the preserved hemisphere following ischemic stroke, but limitations need be considered. First, our observation in this patient would be strengthened by corroboration in a larger population of similar patients, especially considering that subject-level resting-state fMRI measures can be noisy, and we are planning a study to do just that. Second, as we do not have pre-stroke resting-state fMRI in the patient, we cannot rule out that his relatively elevated connectivity pattern was already present prior to the stroke event, although this appears unlikely. Third, the healthy control group was, on average, somewhat younger than the patient and included women. Previous studies in large samples have reported mixed results for the association between age and between-network connectivity (16). The strength of, e.g., Default Mode connectivity appears to follow an inverse U-shape and women exhibit stronger intra-network connectivity compared to men (17). Although it is not clear that these discrepancies bias our result, this should be kept in mind. Finally, we acquired resting-state fMRI session 5 years after the initial stoke event and subsequent treatments. Longitudinal scan sessions in such a patient would provide a clearer approximation of how distributed connectivity patterns change over time, following the stroke event. With these limitations in mind, we view our findings as intriguing but to be interpreted with caution.

In conclusion, future studies are warranted to assess the role of RSFC, including temporal dynamics, and functional recovery in people with surgical and non-surgical hemispherectomy (including dominant and non-dominant hemispheres). This might offer novel avenues for neurorehabilitation. In addition, investigations of RSFC in hemispherectomized people might unravel the functional neuroplasticity that allows the adult brain to preserve full consciousness despite loss of almost half its tissue.

## Data Availability Statement

The raw data supporting the conclusions of this article will be made available by the authors, without undue reservation.

## Ethics Statement

This study was performed as part of the CONNECT-ME study (ClinicalTrials.gov Identifier: NCT02644265) (4). CONNECTME is approved by The Danish Data Protection Agency (RH-2016-191, I-Suite nr: 04760) and the Ethics Committee of the Capital Region of Denmark (Journal-nr.: H-16040845). The patients/participants provided their written informed consent to participate in this study.

## Author Contributions

PF: methodology, software, formal analysis, resources, data curation, writing—original draft, and visualization. SA and VN: methodology, investigation, and writing—original draft. MA: investigation and writing—review and editing. DK: conceptualization, methodology, resources, writing—review and editing and supervision. All authors contributed to the article and approved the submitted version.

## Funding

DK received funding from Rigshospitalets Forskningspulje R143-A6132-B3632, Region Hovedstadens Forskningsfond til Sundhedsforskning 2019 - A6597, Savværksejer Jeppe Juhl og Hustru Ovita Juhls Mindelegat 27062019, Jascha Fonden 2019 - 7684, Offerfonden 19-610- 00060, and the Lundbeck Foundation R349-2020-658. SA received funding from Rigshospitalets Forskningspulje. The funders had no role in study design, data collection and analysis, decision to publish, or preparation of the manuscript.

## Conflict of Interest

The authors declare that the research was conducted in the absence of any commercial or financial relationships that could be construed as a potential conflict of interest.

## Publisher's Note

All claims expressed in this article are solely those of the authors and do not necessarily represent those of their affiliated organizations, or those of the publisher, the editors and the reviewers. Any product that may be evaluated in this article, or claim that may be made by its manufacturer, is not guaranteed or endorsed by the publisher.

## Abbreviations

MCA: Middle Cerebral Artery
fMRI: functional Magnetic Resonance Imaging
rs-fMRI: resting-state fMRI
BOLD: Blood-Oxygen Level Dependent
RSFC: Resting-State Functional Connectivity
NIHSS: National Institutes of Health Stroke Scale
ROIs: Regions Of Interest.
