# Supplementary figures and images for: Case Report: Resting-State Brain-Networks After Near-Complete Hemispherectomy in Adulthood
Source: Front Neurol. 2022 Jun 10;13:885115. doi: 10.3389/fneur.2022.885115 (PMC9226565; doi:10.3389/fneur.2022.885115)

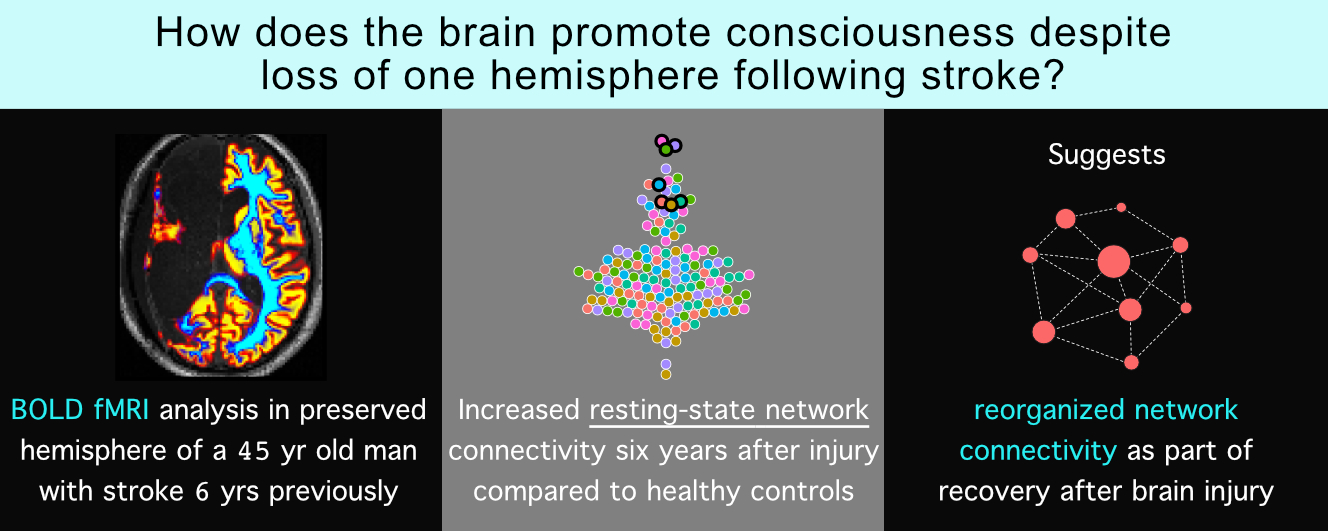

Supplement: Supplementary file 1 [file Image_1.JPEG]
